# Supplementary material for: Active site specificity profiling datasets of matrix metalloproteinases (MMPs) 1, 2, 3, 7, 8, 9, 12, 13 and 14
Source: Data Brief. 2016 Feb 22;7:299–310. doi: 10.1016/j.dib.2016.02.036 (PMC4777984; doi:10.1016/j.dib.2016.02.036)
Supplement: Supplementary file 10 — Supplementary material [file mmc10.zip › WebPICS_hMMP1_G_1%/P1.html]

 

PICS results


|  |  |
| --- | --- |
| **P1\_A**  11 in 126 sites   8.7 %    effects > 10 perc. pnts.  (vice-versa in brackets)  P3\_V: 14.6 (10.1)   P2\_K: 11.9 (16.3)   P2\_N: 13.4 (24.6)   P2prime\_F: 13.4 (24.6)   P2prime\_L: 23.7 (16.3)   P3prime\_K: 10.3 (11.3) |  |
| **P1\_C**  5 in 126 sites   4.0 %    effects > 10 perc. pnts.  (vice-versa in brackets)  P2\_N: 15.2 (12.7)   P3prime\_A: 44.1 (11.0) |  |
| **P1\_H**  7 in 126 sites   5.6 %    effects > 10 perc. pnts.  (vice-versa in brackets)  P3\_V: 30.2 (13.2)   P2\_C: 11.1 (19.4)   P2prime\_K: 38.8 (11.8)   P2prime\_Q: 10.3 (14.4)   P3prime\_K: 49.2 (34.4)   P3prime\_M: 11.1 (19.4) |  |
| **P1\_N**  24 in 126 sites   19.0 %    effects > 10 perc. pnts.  (vice-versa in brackets)  P3\_P: -11.5 (-11.0)   P2\_F: 13.9 (23.9) |  |
| **P1\_P**  6 in 126 sites   4.8 %    effects > 10 perc. pnts.  (vice-versa in brackets)  P2\_L: 54.0 (20.2)   P2prime\_V: 23.0 (10.6)   P2prime\_Y: 13.5 (20.2) |  |
| **P1\_R**  9 in 126 sites   7.1 %    effects > 10 perc. pnts.  (vice-versa in brackets)  P3\_T: 13.5 (11.1)   P3prime\_N: 13.5 (11.1)   P3prime\_Q: 13.5 (11.1) |  |
| **P1\_S**  19 in 126 sites   15.1 %    effects > 10 perc. pnts.  (vice-versa in brackets)  P2\_Y: 11.6 (18.2)   P1prime\_C: 14.8 (34.9)   P1prime\_I: -14.5 (-11.1)   P2prime\_V: 10.8 (15.7) |  |
| **P1\_Y**  7 in 126 sites   5.6 %    effects > 10 perc. pnts.  (vice-versa in brackets)  P3\_A: 38.1 (11.1)   P2\_Q: 46.0 (23.0)   P2prime\_L: 30.2 (13.2)   P3prime\_M: 25.4 (44.4)   P3prime\_Q: 34.2 (21.7) |  |
